# OPTCON3: An Active Learning Control Algorithm for Nonlinear Quadratic Stochastic Problems

**DOI:** 10.1007/s10614-019-09949-0

**Published:** 2019-12-09

**Authors:** V. Blueschke-Nikolaeva, D. Blueschke, R. Neck

**Affiliations:** Department of Economics, University of Klagenfurt, Klagenfurt, Austria

**Keywords:** Stochastic optimal control, Active learning, Dual control, Algorithms

## Abstract

In this paper, we describe the new OPTCON3 algorithm, which serves to determine approximately optimal policies for stochastic control problems with a quadratic objective function and nonlinear dynamic models. It includes active learning and the dual effect of optimizing policies, whereby optimal policies are used to learn about the stochastics of the dynamic system in addition to their immediate effect on the performance of the system. The OPTCON3 algorithm approximates the nonlinear model with a time-varying linear model and applies a procedure similar to that of Kendrick to the series of linearized models to calculate approximately optimal policies. The results for two simple economic models serve to test the OPTCON3 algorithm and compare it to previous solutions of the stochastic control problem. Initial evaluations show that the OPTCON3 approach may be promising to enhance our understanding of the adaptive economic policy problem under uncertainty.

## Introduction

When determining economic policies over some planning horizon, governments and other policy makers are confronted with the problem of uncertainty, both of the effects of their measures on the politically relevant variables and of the trade-offs between different objective variables. Hence such plans should take into account the stochastic nature of the planner’s decision problem, in particular the uncertainty surrounding the relations between different variables which are reflected in the probability distributions of the parameters of the econometric (or calibrated) model of the economy. Stochastic optimal control theory is therefore an appropriate framework to deal with such policy problems when the policy maker’s aim is to obtain the best policy according to his/her preferences.

Unfortunately, stochastic optimal control theory has not succeeded in deriving precise solutions for even very simple analytical problems and even less so for the complex problems involving large models which are characterized by nonlinearities and various sources of uncertainty. One of the reasons for this is the so-called dual effect of controls in a stochastic dynamic system: controls do not only serve to optimize the instantaneous objective in each period but may also be used to learn about the reactions of the economy to policy measures, which in turn can contribute to improved policies in later periods. This interdependence between considerations of direct optimization and experimentation to learn about policy effects makes the stochastic optimal control problem intractable, as has been recognized by several authors in the past (Fel’dbaum 1965; Aoki 1989). One is therefore restricted to numerical investigations determining approximations to the unobtainable truly optimal policies.

So far, the most ambitious work on optimal stochastic control for economic policy problems has been done by Kendrick (1981), who developed several algorithms, including one for active learning, based on Bar-Shalom and Tse (1976), in which the dual effect of controls is explicitly taken into account. Further work with these algorithms revealed interesting problems, such as the occurrence of nonconvexities in linear–quadratic stochastic control problems under active learning (Mizrach 1991; Amman and Kendrick 1995; Tucci 1998; Amman et al. 2018). So far, these algorithms have been confined to linear dynamic models, which is a severe restriction as even the simplest econometric models contain some nonlinearities. In this paper, we extend the Kendrick algorithm with active learning to a class of nonlinear models which can be approximated by time-varying linear models. We first review previous research with the OPTCON algorithms (versions OPTCON1 and OPTCON2) and then present the new OPTCON3 algorithm which includes active learning. Initial evaluations show that this approach may be promising to enhance our understanding of the adaptive economic policy problem under uncertainty.

## The Problem

The OPTCON algorithms are designed to achieve approximate solutions to optimal control problems with a quadratic objective function (a loss function to be minimized) and a nonlinear multivariate discrete-time dynamic system under additive and parameter uncertainties. The intertemporal objective function is formulated in quadratic tracking form, which is quite often used in applications of optimal control theory to econometric models.

Thus, it is required to find values for the control variables $(u_t)$ and the corresponding state variables $(x_t)$ which minimize the function

1 $$\begin{aligned} J=E \left[ \sum ^T_{t=1}L_t(x_t,u_t) \right] , \end{aligned}$$

with

2 $$\begin{aligned} L_t(x_t,u_t)=\frac{1}{2}\left( \begin{array}{c} x_t-\tilde{x}_t\\ u_t-\tilde{u}_t\\ \end{array} \right) 'W_t\left( \begin{array}{c} x_t-\tilde{x}_t\\ u_t-\tilde{u}_t\\ \end{array} \right) \end{aligned}$$

and satisfy conditions in the form of a dynamic system of nonlinear difference equations:

3 $$\begin{aligned} x_t=f(x_{t-1},x_t, u_t, \theta , z_t)+\varepsilon _t, t=1, \ldots , T. \end{aligned}$$

$x_t$ is an *n*-dimensional vector of state variables that describes the state of the economic system at any point in time *t*. $u_t$ is an *m*-dimensional vector of control variables, $\tilde{x}_t\in R^n$ and $\tilde{u}_t\in R^m$ are given ‘ideal’ (desired, target) levels of the state and control variables respectively. *T* denotes the terminal time period of the finite planning horizon. $W_t$ is an $((n+m)\times (n+m))$ matrix, specifying the relative weights of the state and control variables in the objective function. Quite often, $W_t$ is a matrix including a discount factor $\alpha$ with $W_t=\alpha ^{t-1}W$. $W_t$ (or *W*) is symmetric.

Moreover, $\theta$ is a *p*-dimensional vector of parameters whose values are assumed to be constant but unknown to the decision maker (parameter uncertainty), $z_t$ denotes an *l*-dimensional vector of non-controlled exogenous variables, and $\varepsilon _t$ is an *n*-dimensional vector of additive disturbances (system error). $\theta$ and $\varepsilon _t$ are assumed to be independent random vectors with expectations ${\hat{\theta }}$ and $O_n$ respectively and covariance matrices $\varSigma ^{\theta \theta }$ and $\varSigma ^{\varepsilon \varepsilon }$ respectively. *f* is a vector-valued function and $f^i(\ldots )$ is the *i*-th component of $f(\ldots )$, $i=1, \ldots , n$.

## Versions 1 and 2 of the OPTCON Algorithm

This section gives a brief description of the two previous versions of the OPTCON algorithm, with the open-loop and then with the passive learning strategy. The first version of OPTCON, OPTCON1, delivers an open-loop (OL) solution and is described in detail in Matulka and Neck (1992). The open-loop strategy either ignores the stochastics of the system altogether or assumes the stochastics (expectation and covariance matrices of additive and multiplicative disturbances) to be given for all time periods at the beginning of the planning horizon. The problem with the nonlinear system is tackled iteratively, starting with a tentative path of the control and state variables. The tentative path of the control variables is given for the first iteration. In order to find the corresponding tentative path for the state variables, the nonlinear system is solved numerically using the Levenberg-Marquardt method or trust region methods.^1^

**Fig. 1:**
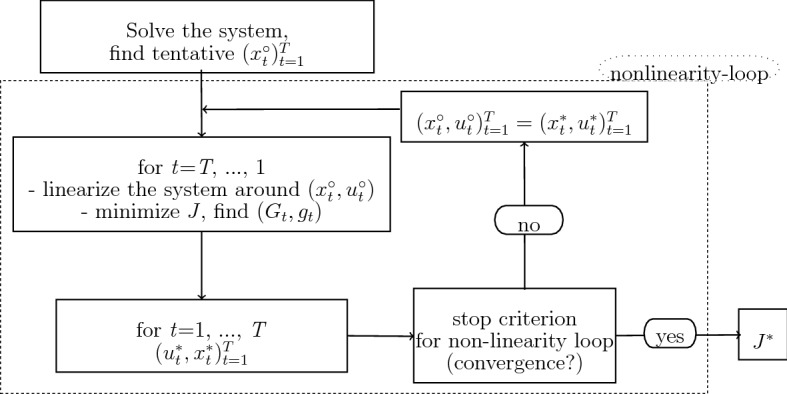
Flow chart of OPTCON1

Then, the iterative approximation of the optimal solution starts. The solution is iterated from one time path to the next until the algorithm converges or the maximum number of iterations is reached. During the optimization process the system is linearized around the previous iteration’s result as a tentative path and the problem is solved for the resulting time-varying linearized system.^2^ The optimal solution of the problem for the linearized system is found under the above-mentioned simplifying assumptions about the information pattern; this solution is then used as the tentative path for the next iteration, starting off the procedure all over again. In every iteration, i.e. for every solution of the problem for the linearized system, the objective function is minimized using Bellman’s principle of optimality to obtain the parameters of the feedback control rule. Finally, the value of the objective function is calculated for the obtained solution. Figure 1 summarizes the OPTCON1 algorithm.

The second version of the algorithm, called OPTCON2 and described in Blueschke-Nikolaeva et al. (2012), includes the passive learning strategy (also named open-loop feedback (OLF)), which uses the idea of re-estimation of the model at the end of each time period. For this re-estimation the model builder (and hence the control agent) observes what has happened and uses the current values of the state variables, that is, the new information, to improve his/her knowledge of the system.

The stochastics in the problem is again represented by two kinds of errors, namely additive (random system errors) and multiplicative (‘structural’ errors in parameters). It is assumed that ‘true’ parameters ${\hat{\theta }}$ generate the model. However, the policy maker does not know these true parameters ${\hat{\theta }}$ and works with the ‘wrong’ parameters $\theta ^m$ resulting from the estimates using the realization of the random variable $\mu ^m$: $\theta ^m = {\hat{\theta }} + \mu ^m$.

The passive learning strategy has the following structure: a forward loop is started from time 1 to *T*. In each time period *S* an (approximately) optimal open-loop solution for the subproblem is determined, i.e the problem for the time periods from *S* to *T*. Then the predicted $x_S^{*}$ and $u_S^{*}$ are fixed for the time period *S*. At the end of each time period the policy maker observes the realized values of the state variables $x_S^{a*}$, which are, however, disturbed by the additive errors. The difference between $x_S^{*}=f(x_{S-1}^{a*},x_S^{*}, u_S^{*}, \theta ^m,z_S)$ and $x_S^{a*}= f(x_{S-1}^{a*}, x_S^{a*},u_S^{*}, {\hat{\theta }},z_S) + \varepsilon _S^m$ comes from the realization of the random numbers $\varepsilon _S^m$ and $\mu ^m$. Next, the new information is used by the policy maker to update and adjust the parameter estimate $\theta ^m$. After that, the same procedure is applied to the remaining subproblem from $S+1$ to *T*, and so on. The update of the parameter estimates is conducted via the Kalman Filter.

The same update procedure is used in the next version of the OPTCON algorithm (which is called OPTCON3) as well.

## The OPTCON3 Algorithm

### Description

The new version of the OPTCON algorithm includes an active learning strategy (also called closed-loop, adaptive dual or dual control) and is named OPTCON3. The active learning strategy lets the policy maker face the dual problem of choosing the best strategy and reducing the uncertainty about the system. It is expected that such a strategy can help improve the performance of the control process and give more reliable policy recommendations. The active learning method differs from the passive learning method in the OPTCON2 algorithm in the following way. When using the passive learning method, new observations are obtained each period and are used to update the parameter estimates; however, no effort is made to choose control variables with the aim of improving the learning process about the dynamic system to be controlled. In contrast, in the active learning methods, control variables are chosen with the dual purpose of moving the system in the desired direction and perturbing the system to improve the parameter estimates. Thus, the active learning strategy delivers an optimal solution where the control is chosen with a view to reaching the desired states in the present and reducing uncertainty through learning, permitting an easier attainment of desired states in the future. This lets the policy maker cope with the dual problem of choosing the best strategy and reducing the uncertainty about the system simultaneously. The key idea is to make some use of information about future measurements as well.

The procedure of finding the closed-loop solution in this paper corresponds to Kendrick (1981). The approximate cost-to-go is broken down into three terms: $J_d=J_{D}+J_{C}+J_{P}$, where $J_d$ is the total cost-to-go with *T* periods remaining; the deterministic component $J_D$ includes only non-stochastic terms; the cautionary component $J_{C}$ includes the stochastic component of the system known in the current period; and the probing term $J_P$ contains the effect of dual learning on the future time periods. Each of these components faces special difficulties in computing due to the nonlinearity of the system. Especially the probing term includes the motivation to perturb the controls in the present time period in order to reduce future uncertainty about the parameter values and can therefore be considered the most challenging task. Thus, the terms $J_{C}$ and $J_P$ constitute a separate optimization problem with a quadratic criterion which is maximized subject to the nonlinear system. The system equations are derived from the expansion of the original system and can be calculated by rewriting the Taylor expansion of the nonlinear system in the perturbation form $\delta x_t$. Instead of the system (3) the objective function in perturbation form has to be solved: $min_{\delta u_t} \varDelta J^*_{t}$. After some calculations the solution $\varDelta J^*_{t}$ is presented in quadratic form as a function of $\delta x_{t-1}$. The original $J^*_{t}$ can be derived from $\varDelta J^*_{t}$ and can be decomposed in three terms. Moreover, all the terms and formulas need to be adjusted to the augmented system $$\left[ \begin{array}{c} x_t \\ \ldots \ldots \\ \theta \\ \end{array} \right] $$.^3^

**Fig. 2:**
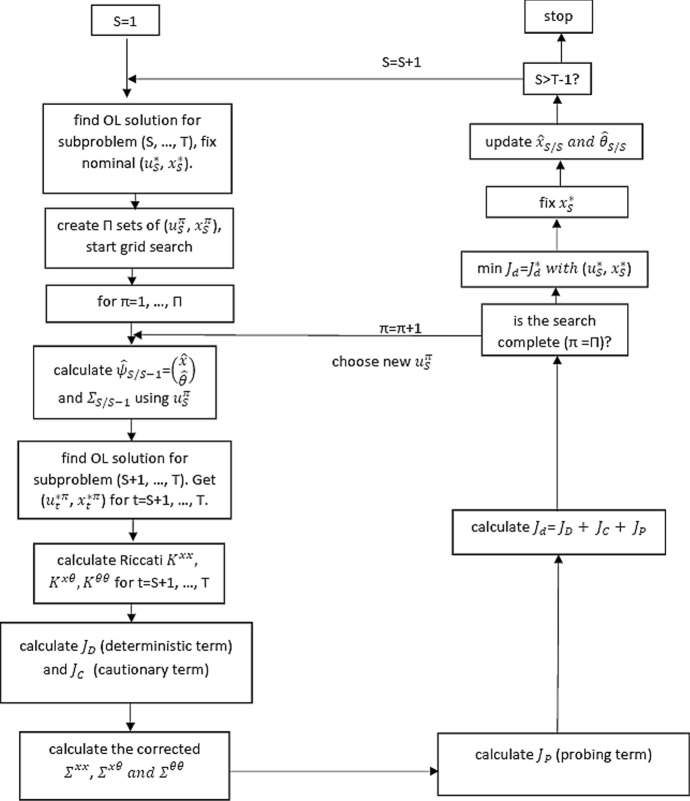
Flow chart of OPTCON3

Next, a schematic structure of the OPTCON3 algorithm is presented. This goes in line with the simplified flow chart presented in Fig. 2 and is used as a basic structure for the implementation.

The optimization is carried out in a forward loop from 1 to *T*. In each time period *S* ($S=1,\ldots ,T$) the following search procedure is conducted. The subproblem from *S* to *T* is solved via the open-loop (OL) strategy (see Fig. 1 in Sect. 3). The OL solution of $(x_S^{*}, u_S^{*})$ for the time period *S* is fixed. After that the core part of the dual control starts. The idea is to actively search for some solution paths which best deal with the dual problem of minimizing the current objective function and the future uncertainty in the model. In this paper a grid search method is used.^4^ For this purpose we create a grid of possible solutions around the existing path $(x_S^{*}, u_S^{*})$. We denote the grid search procedure as “$\pi$-loop”. In each iteration ($\pi =1,\ldots ,\varPi$) the approximate objective function is evaluated which corresponds to the search value of the control. The evaluation is repeated until the approximately optimal control is found. Inside the search loop (for each $\pi$) the following steps are to be performed.

An (approximately) optimal open-loop solution for the subproblem (i.e. the problem for the time periods from $S+1$ to *T*) is determined. Then the OL solution $(x_{S+1}^{* \pi }, u_{S+1}^{* \pi })$ for the time period $S+1$ is fixed. Next, after some auxiliary calculations (Riccati matrices) the deterministic, cautionary and probing terms of the cost-to-go are determined. In the process, a new loop is introduced, where the terms $J_{D}$, $J_{C}$ and $J_{P}$ for the time periods $j=S+1,\ldots ,T$ are calculated step by step from time period $S+1$ to *T* using the updated covariances. Once the $\pi$-loop has been completed, the total approximate objective function $J_d=J_D+J_C+J_P$ can be obtained. The evaluation of the function is done at each iteration in the $\pi$-loop. When the search is completed, i.e. the approximately optimal path with $min J_d$ is found, the new information is used by the policy maker to update and to adjust the parameter estimate $\theta ^m$, whereby the Kalman filter is used. After that, the same procedure is applied for the remaining subproblems from $S+2$ to *T*, and so on.^5^

The OPTCON3 algorithm essentially uses the approach introduced by Bar-Shalom and Tse (1976) and Kendrick (1981) but augments it by approximating, in each step, the nonlinear system by a series of linear systems (replacing the nonlinear autonomous system by a linear time-varying one).

The OPTCON3 algorithm (Steps I - IV in the appendix) describes the steps how to obtain an approximately optimal dual control solution of a stochastic problem. In the optimization process one has to observe the current state of the system, which is crucial for the learning procedure. Because it is not possible to observe current and true values for a performance test, one has to resort to Monte-Carlo simulations. In this way, some “quasi-real” values can be created and used to compare the performance of an optimization without learning (both open-loop (OL) and certainty equivalence (CE) alternatives), passive learning (OLF) and active learning (AL).

Thus, a large number *M* (a number, usually between 100 and 1000) of realizations of random noises $(\varepsilon _t^m)_{t=1}^T$ and $\mu ^m$, $m = 1, \ldots , M$, are generated. It is assumed that there is an unknown ‘real’ model with the ‘true’ constant parameter vector ${\hat{\theta }}$. But the policy maker does not know these ‘true’ parameters ${\hat{\theta }}$ and works with the ‘wrong’ parameters $\theta ^m$ resulting from the estimates using the realization of the random variable $\mu ^m$: $\theta ^m = {\hat{\theta }} + \mu ^m$. For better understanding, a brief scheme is sketched in Algorithm 1.
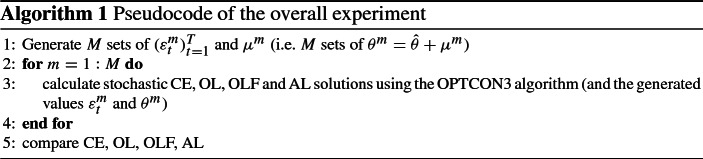


Algorithm 1 is used in the next section to test the performance of the new algorithm.

## Applications

We apply Algorithm 1 to two different models and test the performance of active learning in terms of the objective function value and influence on the control variable. In a simple linear model, the MacRae model, we observe a relatively small effect of active learning. In contrast, in a more sophisticated model ATOPT, using the active learning strategy leads to a more active use of the control variable.

*The MacRae model*

The MacRae model, as used by MacRae (1972) and Kendrick (1981), is a theoretical model for two periods only. The MacRae model includes one control variable and one state variable and consists of one equation only:

4 $$\begin{aligned} \begin{array}{ccccccccccccc} x_t &{} = 3.5 &{} + &{} 0.7 &{} x_{t-1} &{} - &{} 0.5 &{} u_t &{} + &{} \varepsilon _t, &{} &{}x_0=0\\ &{} &{} &{} &{} &{} &{} (0.5) &{} &{} &{} &{} \\ \end{array} \end{aligned}$$

The model does not have exogenous (non-controlled) variables. One of the parameters is treated as unknown.^6^ The objective function penalizes deviations of objective variables from their target values. The target values of the state and control variables ($\tilde{x}_t$ and $\tilde{u}_t$ respectively) are assumed to be zero. The weight matrix *W* is assumed to be constant over time (no discounting). The weights for the state and the control variables (the values in *W*) are chosen to be 1, which reflects the same importance for all variables. The optimization horizon consists of 2 periods.

**Fig. 3:**
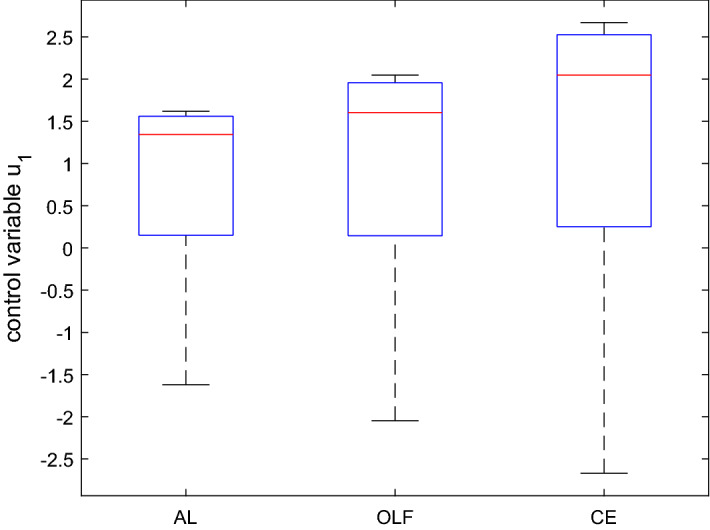
Boxplot for the control variable in $t=1$ based on a Monte Carlo experiment with 1000 draws, MacRae model

The aim of the application is to determine approximately optimal policies under the assumed objective function and the dynamic system given here by Eq. (4) using the three versions of the OPTCON algorithm, i.e. the three strategies: certainty equivalence (CE), open-loop feedback (OLF) and active learning (AL). Figure 3 summarizes, in the form of a boxplot, the optimization results, i.e. the optimal values of the control variable in different Monte Carlo runs for all three strategies.

In the boxplot in Fig. 3, $50 \%$ of all scores are placed in the boxes and the median is shown by the line that divides the box into two parts. It shows that the results of the OLF strategy are more concentrated (the OLF box is smaller) than those of CE. The results of the AL strategy are even more concentrated than those of CE and OLF. This is to some extent due to the simplicity of the model and the fact that there is just one optimization period for active learning.

We can also observe the following: CE has the minimum cost in approximately $66\%$ of the cases, AL in $22\%$ and OLF in remaining cases. This may be compared to the results in Kendrick’s DUAL code, which are $60\%$, $25\%$, and $15\%$, respectively.

**Fig. 4:**
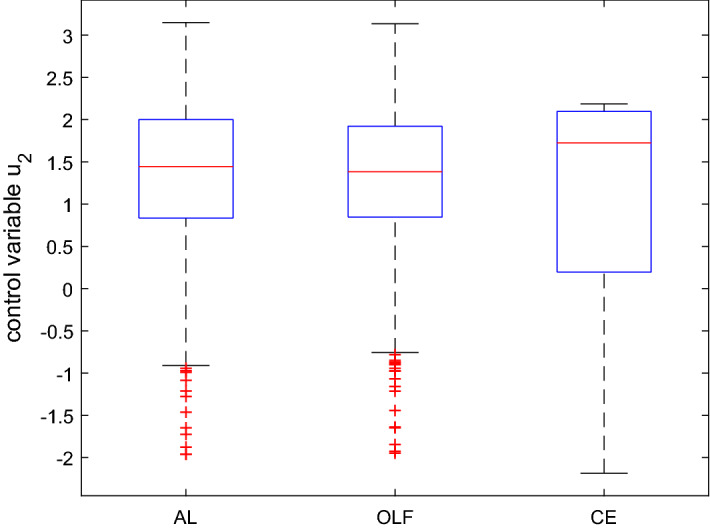
Boxplot for the control variable in $t=2$ based on a Monte Carlo experiment with 1000 draws, MacRae model

The mean and the standard deviation of the optimal values of the objective function are given by $mean(J^{OLF}) = 20.11$, $mean(J^{AL}) = 20.18$, $mean(J^{CE}) = 22.18$, and $std(J^{OLF}) = 6.35$, $std(J^{AL}) = 5.93$, $std(J^{CE}) = 11.36$, respectively. As with Kendrick’s DUAL software, the difference in the average cost of the three procedures is rather small and the AL algorithm gives the minimum standard deviation, whereas the avg. cost of CE has the highest standard deviation, with larger differences than for the mean. In particular, Fig. 3 suggests that the control associated with AL for $t = 1$ is less active than the others, almost half so of the CE control, and its standard deviation is also much lower than all the others. From Fig. 4, we see that the differences in the controls and standard deviations are less pronounced for the final period. Especially the results for OLF and AL are very similar.

**Fig. 5:**
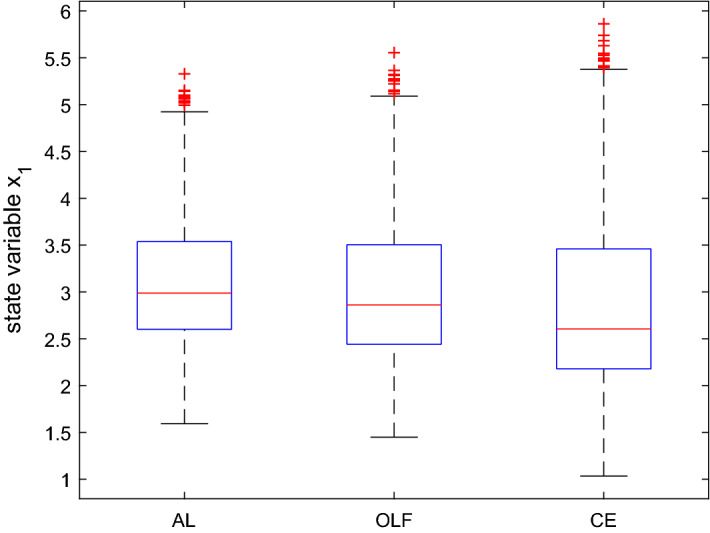
Boxplot for the state variable in $t=1$ based on a Monte Carlo experiment with 1000 draws, MacRae model

**Fig. 6:**
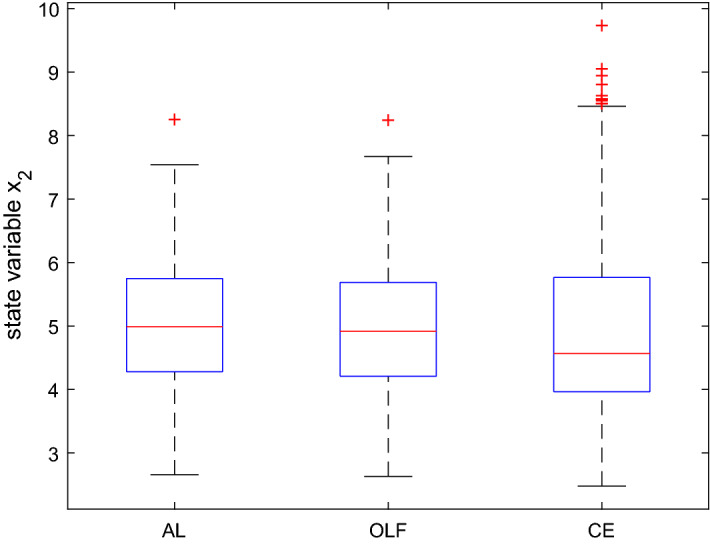
Boxplot for the state variable in $t=2$ based on a Monte Carlo experiment with 1000 draws, MacRae model

As far as the state is concerned (Figs. 5 and 6), all three procedures show similar results at the end of the time horizon. AL performs slightly better than the others at $t = 1$. Again, standard deviations are lower when using AL. However, the differences are much smaller as compared to the control variable. Summarizing, by and large we can confirm the results from Kendrick’s DUAL code.

**Fig. 7:**
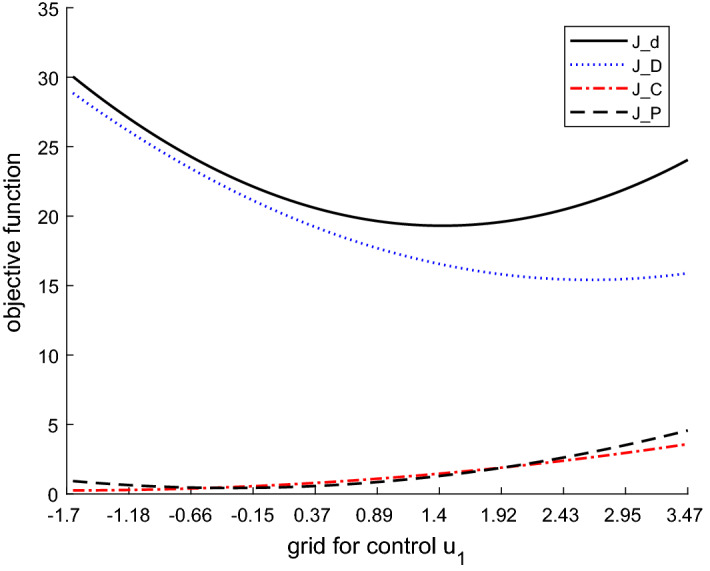
Components of the objective function based on a Monte Carlo experiment with 100 draws, MacRae model

Figure 7 shows the values of the three components of the objective function, deterministic ($J_D$), cautionary ($J_C$) and probing terms ($J_P$), and the total objective function ($J_d$) [see Eqs. (13), (14), (17)].

The deterministic component contributes most to the values of the objective function $J_d$. The values of the cautionary and probing terms are much smaller. The deterministic cost component falls with increases in the control values and the other two components rise. Moreover, the probing component delivers the smallest part of the total cost. These results are due to the simplicity of the model, in particular its linearity. They are similar to those obtained by Kendrick (1981) and Kendrick (1982). Thus, an application to a more sophisticated, nonlinear model will be carried out in the next step.

*The ATOPT model*

Next, we apply the algorithm to a nonlinear dynamic model of the Austrian economy (ATOPT model) created by Blueschke et al. (2018), which analyzes the output – public debt trade-off. The model consists of three equations, i.e. three endogenous variables: output growth $(y_t)$, public debt $(d_t)$, and the interest rate $(r_t)$, which are:

5 $$\begin{aligned} y_t= & {} a_1\cdot y^{world}_t - \theta _1 \cdot g_t + \varepsilon _{1,t} \end{aligned}$$

6 $$\begin{aligned} d_t= & {} (1 + r_t) \cdot d_{t-1} - g_t + \varepsilon _{2,t} \end{aligned}$$

7 $$\begin{aligned} r_t= & {} r_{t-1} + \theta _2\cdot (y_t-{\bar{y}}_t) + a_2\cdot (d_t - {\bar{d}}_t)^3 + \varepsilon _{3,t} \end{aligned}$$

**Fig. 8:**
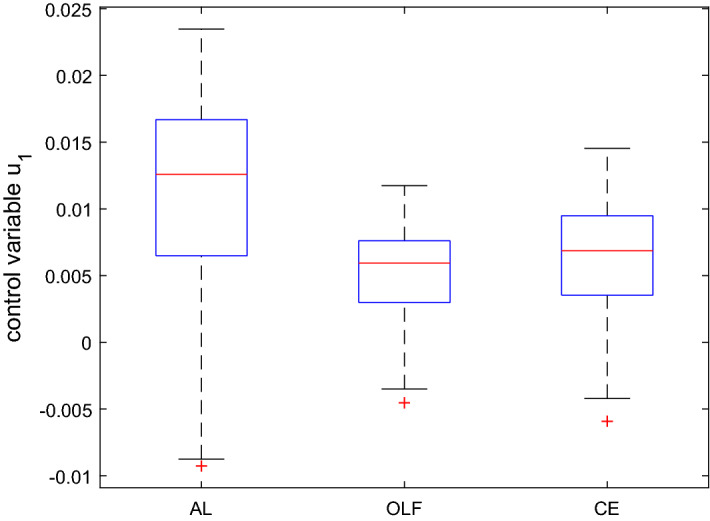
Boxplot for the control variable in $t=1$ based on a Monte Carlo experiment with 100 draws, ATOPT model

Austria is a small open economy; thus its economic performance depends to a large extent on the economic situation in the world. The correlation coefficient ($a_1$) between Austria’s and the world’s GDP growth (between 1996 and 2017) is 0.7266. The fiscal policy instrument ($g_t$) is the primary fiscal surplus (or deficit if negative). For the Austrian economy, an acceptable debt level is assumed to be given by the Maastricht criterion of 60% of GDP. As a threshold for normal output growth, a value slightly above the historical average (1996–2017) of 1.84 is assumed here, namely 2 percent annual growth (${\bar{y}} = 0.02$). The fiscal multiplier parameter ($\theta _1$) is one of the two stochastic parameters in the model and is assumed to be 1.2 with variance $\varSigma ^{\theta _1}=0.5$. The second stochastic parameter ($\theta _2$) is the link between output growth and the interest rate and is equal to 0.1 with variance $\varSigma ^{\theta _2}$=  0.1.

Equations (5)–(7) give a very simplified description of the Austrian economy with an output growth—public debt trade-off. Using its instrument, namely fiscal policy *g*, the government aims at maintaining a high GDP growth of 3% ($\tilde{y}=0.03$) and a steady decrease in public debt from 78.4% of GDP in 2017 to 60% of GDP at the end of the planing horizon, namely in 2022 ($T=5$). At the same time, the government prefers to have a balanced budget ($\tilde{g}=0$). The former two targets are represented by state variables in the ATOPT model, while the latter objective variable is the control variable.

**Fig. 9:**
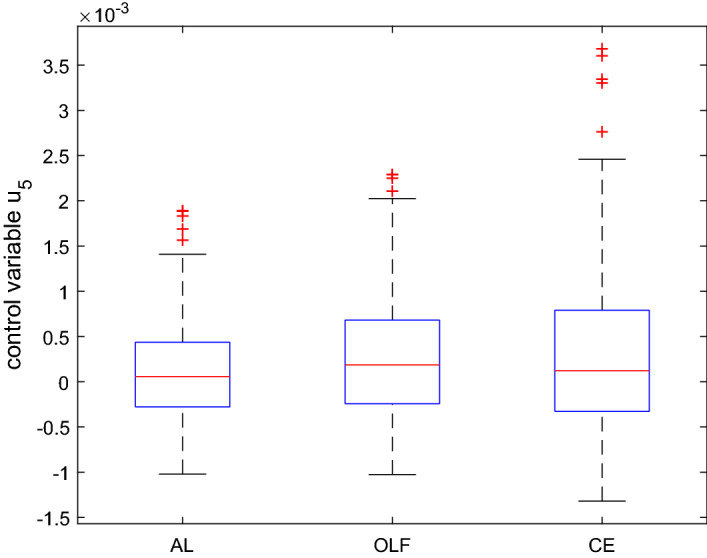
Boxplot for the control variable in $t=5$ based on a Monte Carlo experiment with 100 draws, ATOPT model

**Fig. 10:**
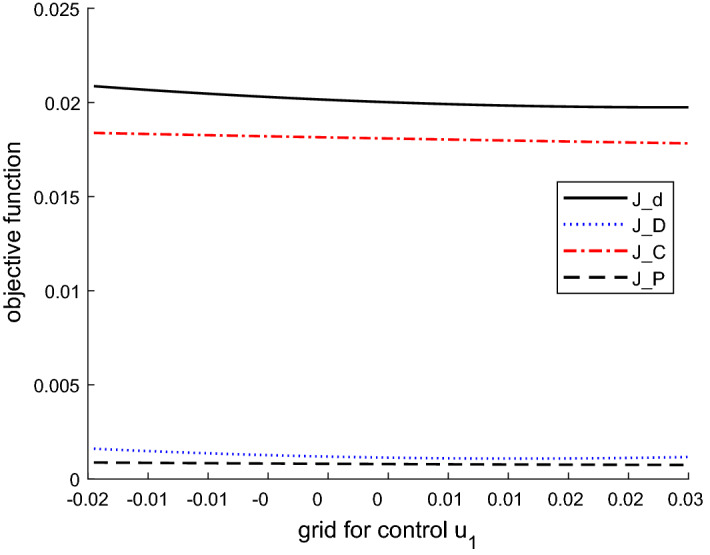
Objective function values based on a Monte Carlo experiment with 100 draws, ATOPT model

Thus, the task is to find an optimal path for the control variable, in order to minimize the sum of the squared differences between the outcome of the system and the given targets. The optimal control problem is solved again using CE, OLF and AL strategies over the time horizon 2018–2022. The results are shown in Figs.  8, 9 and 10. The box plot in Fig. 8 illustrates the values of the first control variable in the first time period and the box plot in Fig. 9 shows the same control variable in period 5.

We see that in period 1 the AL strategy allows for a lot of variation or probing. This is in line with the idea of active learning. As a result of this probing the AL strategy delivers much better results in time period 5 than the other two strategies. The AL results of different MC runs are not spread out as much as CE and OLF in the last period. This qualitative behaviour of the active-learning control (relatively strong variations at the beginning to elicit reactions from the system from which to learn and to get closer to the “true” system parameters at the end) has also been observed by Kendrick (1982) in a linear model.

In Fig. 10 we can see that in contrast to the MacRae problem (Fig. 7), the cautionary component is much bigger and the deterministic term is smaller. Thus the largest part of the total objective function is due to the cautionary term. The explanation is that here we have a more complex optimization problem (compared to the MacRae problem) and, in particular, optimization over a longer planning horizon. These insights are in line with the results in Kendrick (1981).

## Conclusion

In this paper, we reviewed the algorithms OPTCON1 for open-loop and OPTCON2 for open-loop feedback (passive learning) control for stochastic economic policy models and describe in detail the new OPTCON3 algorithm, which includes active learning and the dual effect of optimizing policies. The OPTCON algorithms are applicable to nonlinear models which can be approximated by time-varying linear models. A computer program was created to implement approximately optimal policies according to the OPTCON3 algorithm. The results from calculating these policies for two simple economic models served to test the OPTCON3 algorithm and compare it to the previous solutions of the stochastic control problem. Initial evaluations show that the OPTCON3 approach may be promising to enhance our understanding of the adaptive economic policy problem under uncertainty.

## Appendix

**The detailed description of the steps of the OPTCON3 algorithm.**

The following values are given as input:

$$f(\ldots ) $$: system function
$x_{0}=\overset{\circ }{x}_{0}$: initial values of state variables
$(\overset{\circ }{u}_t)_{t=1}^T$: tentative path of control variables
${\hat{\theta }}_{0}={\hat{\theta }}$: expected values of system parameters
$\varSigma ^{\theta \theta }_{0}=\varSigma ^{\theta \theta }$: covariance matrix of system parameters
$\varSigma ^{\varepsilon \varepsilon }$: covariance matrix of system noise
$\theta ^m$: (random) parameter noises
$(\varepsilon ^m_t)_{t=1}^T$: (random) system noises
$(z_t)_{t=1}^T$: path of exogenous variables
$(\tilde{u}_t)_{t=1}^T$: target path for control variables
$(\tilde{x}_t)_{t=1}^T$: target path for state variables
$W^{xx}$, $W^{ux}$, $W^{uu}$: weighting matrices of objective function
$\alpha$: discount rate of objective function

At the end of the algorithm the following optimal values have to be obtained: $(x_t^{a*})_{t=1}^T$, $(u_t^*)_{t=1}^T$ and $J^*$.

*Step I:* For each *S* from 1 to *T* do the following search steps [1]-[3]:

*Step I-1:* Find an open-loop solution for the subproblem $(S,\ldots ,T)$: apply the procedure already implemented in OPTCON1; cf. Sect. 3 above. Fix $(x_S^*, u_S^*)$.

*Step I-2:* Run a grid search of size $\varPi$ around $(x_S^*, u_S^*)$, i.e., for each $\pi =1, \ldots , \varPi$ perform the steps (A) - (G):

*Step I-2A:* Find the open-loop solution $(x^{*}_t, u^{*}_t)_{S+1}^T$ for the subproblem ($S+1$, ..., *T*).

$$\bullet $$ The *nonlinearity loop* is run until the stop criterion is fulfilled, i.e. until the difference between the values of the current and the previous iteration is smaller than a pre-specified number or the maximum number of iterations is achieved. The approximately optimal solution $$(x^{*}_t, u^{*}_t)_{S+1}^T$$ has been found when the stop criterion has been achieved. Then go to the next step **I-2B**. It should be noted that after several runs of the nonlinearity loop only the solution ($$x^{*}_{S+1}, u^{*}_{S+1})$$ for the time period $$S+1$$ will be taken as the optimal (nominal) solution. The calculations of the pairs $$(x^{*}_{t'}, u^{*}_{t'})$$ for other periods ($$t'$$ > $$S+1$$) have to be done again, taking into account the re-estimated parameters for all periods.

Notice the parameter matrices for the linearized system of equations: $A=(I-F^x_{x_t})^{-1}F^x_{x_{t-1}}$ and $B=(I-F^x_{x_t})^{-1}F^x_{u_{t}}$. $F^x_{x_t}$, $F^x_{x_{t-1}}$ and $F^x_{u_{t}}$ are the derivatives of the system function $f(\ldots )$ with respect to $x_t$, $x_{t-1}$ and $u_t$ respectively.^7^

*Step I-2B:* Calculate the Riccati matrices $K^{xx}$, $K^{x\theta }$ and $K^{\theta \theta }$ and the auxiliary matrices $\varLambda ^{xx}$, $\varLambda ^{xu}$, $\varLambda ^{uu}$, $\lambda ^{x}$ and $\lambda _t^{u}$ for time periods $t=S+1, \ldots , T$.^8^

The initialization for backward recursion has to be done:

9 $$\begin{aligned} H_{T+1}^{xx}= & {} O_{n\times n}, h^x_{T+1}=O_{n},\nonumber \\ H_{T+1}^{x\theta }= & {} O_{n\times l}, H_{T+1}^{\theta \theta }=O_{l\times l}.\nonumber \\ K_t^{xx}= & {} W^{xx}_t+H^{xx}_{t+1}, K_t^{\theta x}=H_{t+1}^{\theta x}, K_t^{\theta \theta }=H_{t+1}^{\theta \theta },\nonumber \\ k_t^x= & {} h_{t+1}+x'W^{xx}_t+W^{xu}_t u+w_t^x \end{aligned}$$

10 $$\begin{aligned} \varLambda _t^{xx}= & {} (A_t)'K^{xx}_tA_t\nonumber \\ \varLambda _t^{ux}= & {} (B_t)'K^{xx}_tA_t+W_t^{ux}A_t\nonumber \\ \varLambda _t^{xu}= & {} (\varLambda _t^{ux})'\nonumber \\ \varLambda _t^{uu}= & {} (B_t)'K^{xx}_tB_t+2(B_t)'W_t^{xu}+W_t^{uu} \end{aligned}$$

11 $$\begin{aligned} \lambda _t^{x}= & {} (A_t)'k_t^x \nonumber \\ \lambda _t^{u}= & {} (B_t)'k_t^x+x'W^{xu}_t+W^{uu}_t u+w_t^u \end{aligned}$$

*Step I-2C:* Compute the parameters: $H^{xx}$, $H^{x\theta }$, $H^{\theta \theta }$ and $h^x$ for $t=S+1, \ldots , T$:

12 $$\begin{aligned} H_t^{xx}= & {} \varLambda ^{xx}_t-\varLambda ^{xu}_t(\varLambda ^{uu}_t)^{-1}\varLambda ^{ux}_t,\nonumber \\ H_t^{\theta x}= & {} [D'K_t^{xx}+K_t^{\theta x}]A_t-[[D'K_t^{xx}+K_t^{\theta x}]B_t+DW^{xu}](\varLambda _t^{uu})^{-1} \varLambda _t^{ux}\nonumber \\ H_t^{\theta \theta }= & {} D'(K_t^{xx}D+K_t^{x \theta })+K_t^{\theta x}D+ K_t^{\theta \theta }-[[D'K_t^{xx}+K_t^{\theta x}]B_t+DW^{xu}]\nonumber \\&\quad \times (\varLambda _t^{uu})^{-1} [B_t[K_t^{xx}D+K_t^{x \theta }]+W^{ux}D],\nonumber \\ h_t^x= & {} \lambda ^{x}_t-\varLambda ^{xu}_t(\varLambda ^{uu}_t)^{-1}\lambda ^{u}_t, \end{aligned}$$

where $$D=(I-F^x_{x_t})^{-1}F^x_{\theta }$$.

$F^x_{\theta }$ is the derivative of the system function with respect to $\theta$.

*Step I-2D:* Calculate the deterministic component of the approximate objective function $J_{D,S}$ and the cautionary component $J_{C,S}$.

13 $$\begin{aligned} J_{D,S}= & {} \frac{1}{2}[x^*_{S}-\tilde{x}_S]'W^{xx}[x^*_{S}-\tilde{x}_S]+[x^*_{S}-\tilde{x}_S]'W^{xu}[u^*_S-\tilde{u}_S]\nonumber \\&+\frac{1}{2}[u^*_S-\tilde{u}_S]'W^{uu}[u^*_S-\tilde{u}_S] \text { and }\nonumber \\ J_{C,S}= & {} \frac{1}{2}tr(H^{xx}_{S+1}\varSigma ^{xx}_{S+1/S})+tr(H^{\theta x}_{S+1}\varSigma ^{x \theta }_{S+1/S})+\frac{1}{2}tr(H^{\theta \theta }_{S+1}\varSigma ^{\theta \theta }_{S+1/S}) \end{aligned}$$

*Step I-2E:* Repeat for each *j* ($j=S+1, \ldots , T$) the steps [a]-[c]:

   **[a]:** Calculate the deterministic component $J_{D,j}$ and the cautionary component $J_{C,j}$.

14 $$\begin{aligned} J_{D,j}= & {} \frac{1}{2}[x^*_j-\tilde{x}_j]'W^{xx}[x^*_j-\tilde{x}_j]'+[x^*_j-\tilde{x}_j]'W^{xu}[u^*_j-\tilde{u}_j]\nonumber \\&+\frac{1}{2}[u^*_j-\tilde{u}_j]'W^{uu}[u^*_j-\tilde{u}_j])\nonumber \\ J_{C,j}= & {} \frac{1}{2}tr(K^{xx}_j\varSigma ^{\xi \xi }) \end{aligned}$$

   **[b]:** Calculate the matrix $\varSigma ^{\theta \theta }_{j/j}$.

15 $$\begin{aligned} \varSigma ^{\theta \theta }_{j/j}=\varSigma ^{\theta \theta }_{j/j-1}-\varSigma ^{\theta x}_{j/j-1}(\varSigma ^{xx}_{j/j-1})^{-1}\varSigma ^{x\theta }_{j/j-1} \end{aligned}$$

with

16 $$\begin{aligned} \varSigma ^{xx}_{j/j-1}= & {} F^x_{\theta }\varSigma ^{\theta \theta }_{j-1/j-1}(F^x_{\theta })'+\varSigma ^{\varepsilon \varepsilon }_j \text { and } \nonumber \\ \varSigma ^{x \theta }_{j/j-1}= & {} (\varSigma ^{\theta x}_{j/j-1})'=F^x_{\theta }\varSigma ^{\theta \theta }_{j-1/j-1},\nonumber \\ \varSigma ^{\theta \theta }_{j/j-1}= & {} \varSigma ^{\theta \theta }_{j-1/j-1}. \end{aligned}$$

   **[c]:** Calculate the probing component $J_{P_j}$.

17 $$\begin{aligned} J_{P_{j}}= & {} \frac{1}{2}tr[\varLambda ^{xu}(\varLambda ^{uu})^{-1}\varLambda ^{ux}\varSigma _{j/j}^{xx}]\nonumber \\&+tr[\varLambda ^{xu}(\varLambda ^{uu})^{-1}(B'(K^{xx}D+K^{x \theta })+W^{ux}D)\varSigma _{j/j}^{\theta x}]\nonumber \\&+\frac{1}{2}tr[((K^{\theta x}+D'K^{xx})B+D'W^{xu})(\varLambda ^{uu})^{-1}\nonumber \\&\times (B'(K^{xx}D+K^{x \theta })+W^{ux}D)\varSigma _{j/j}^{\theta \theta }] \end{aligned}$$

*Step I-2F:* Calculate the sum of the deterministic, cautionary and probing terms over the periods $S, \ldots , T$:

$$\begin{aligned} J_d=(J_{D,S}+\sum _{j=S+1}^T J_{D,j})+(J_{C,S}+\sum _{j=S+1}^TJ_{C,j})+\sum _{j=S+1}^TJ_{P_{j}}. \end{aligned}$$

*Step I-2G:* Take a new control $\overset{\circ }{u}_S=\overset{\circ }{u}_S^{\pi +1}$ (new point of the grid search) and go to step I-2A.

*Step I-3:* Choose an optimal $u_S^*$ with $min J=J_d^*(u_S^*)$. End of grid search.

*Step II:* Calculate the following a) and b) for only one time period *S*:

a)

18 $$\begin{aligned} \varSigma ^{xx}_{S/S-1}= & {} F^x_{\theta }\varSigma ^{\theta \theta }_{S-1/S-1}(F^x_{\theta })'+\varSigma ^{\varepsilon \varepsilon }_S \text { and } \nonumber \\ \varSigma ^{x \theta }_{S/S-1}= & {} (\varSigma ^{\theta x}_{S/S-1})'=F^x_{\theta }\varSigma ^{\theta \theta }_{S-1/S-1},\nonumber \\ \varSigma ^{\theta \theta }_{S/S-1}= & {} \varSigma ^{\theta \theta }_{S-S/S-1}. \end{aligned}$$

b)

$$\begin{aligned} x^{a*}_S=f(x^{a*}_{S-1}, x^{a*}_S, u^*_S, {\hat{\theta }}) + \varepsilon _S^m. \end{aligned}$$

*Step III:* Update the parameter estimates $\theta ^m$ and $\varSigma ^{\theta \theta }_{S/S}$:

19 $$\begin{aligned} \theta ^m_{S/S}= & {} \theta ^m_{S/S-1}+\varSigma ^{\theta x}_{S/S-1}(\varSigma ^{xx}_{S/S-1})^{-1}[x^{a*}_{S}-x^*_S] \text { and } {\hat{x}}_{S/S}=x_S^{a*}. \end{aligned}$$

20 $$\begin{aligned} \varSigma ^{\theta \theta }_{S/S}= & {} \varSigma ^{\theta \theta }_{S/S-1}-\varSigma ^{\theta x}_{S/S-1}(\varSigma ^{xx}_{S/S-1})^{-1}\varSigma ^{x\theta }_{S/S-1} \end{aligned}$$

*Step IV:* Set $\theta ^m=\theta ^m_{S/S}$ and $\varSigma ^{\theta \theta }=\varSigma ^{\theta \theta }_{S/S}$, go to Step I and run the procedure for the time period $S+1$. The loop of Step I and the OPTCON3 algorithm is finished when $S=T$ and the approximately optimal dual control and state variables for all periods have been found.

## Footnotes

1 Alternatively, the Newton-Raphson or the Gauss–Seidel method can be applied.

2 The criterion for convergence demands that the difference between the values of current and previous iterations be smaller than a pre-specified number.

3 In this case, $\theta$ is constant over time.

4 The search method may also be some other procedure, such as gradient optimization or one of the heuristic approaches.

5 The detailed description of the steps in the OPTCON3 algorithm is presented in the appendix.

6 The standard deviation of the uncertain parameter is given in brackets below the coefficient.
